# Immune Regulation by Pericytes: Modulating Innate and Adaptive Immunity

**DOI:** 10.3389/fimmu.2016.00480

**Published:** 2016-11-04

**Authors:** Rocío Navarro, Marta Compte, Luis Álvarez-Vallina, Laura Sanz

**Affiliations:** ^1^Molecular Immunology Unit, Hospital Universitario Puerta de Hierro Majadahonda, Madrid, Spain; ^2^Immunotherapy and Cell Engineering Laboratory, Department of Engineering, Aarhus University, Aarhus, Denmark

**Keywords:** pericytes, innate immunity, inflammation, adaptive immunity, tumor microenvironment

## Abstract

Pericytes (PC) are mural cells that surround endothelial cells in small blood vessels. PC have traditionally been credited with structural functions, being essential for vessel maturation and stabilization. However, an accumulating body of evidence suggests that PC also display immune properties. They can respond to a series of pro-inflammatory stimuli and are able to sense different types of danger due to their expression of functional pattern-recognition receptors, contributing to the onset of innate immune responses. In this context, PC not only secrete a variety of chemokines but also overexpress adhesion molecules such as ICAM-1 and VCAM-1 involved in the control of immune cell trafficking across vessel walls. In addition to their role in innate immunity, PC are involved in adaptive immunity. It has been reported that interaction with PC anergizes T cells, which is attributed, at least in part, to the expression of PD-L1. As components of the tumor microenvironment, PC can also modulate the antitumor immune response. However, their role is complex, and further studies will be required to better understand the crosstalk of PC with immune cells in order to consider them as potential therapeutic targets. In any case, PC will be looked at with new eyes by immunologists from now on.

## Introduction

Pericytes (PC) were first described 145 years ago by Carl Joseph Eberth and “rediscovered” 2 years later by Charles-Marie Benjamin Rouget. They were given their current name by Karl Wilhelm Zimmermann in 1923 (1), due to their location wrapping around capillaries and postcapillary venules. However, they remain elusive cells with intriguing properties that have only recently attracted the attention of numerous researchers. This is due, in part, to the relatively low numbers of PC in most tissues (with the CNS being an exception) and exacerbated by the absence of truly unique markers, increasing the difficulty of isolating pure primary PC. In fact, cultured human PC have not been readily available until a few years ago, which explains the sparse data available on PC compared to the far more characterized endothelial cells (EC) (2).

To make the picture more complex, PC are heterogeneous in terms of phenotype, distribution, and embryonic origin. Markers used to identify PC include PDGF receptor-β (PDGFR-β), nerve-glial antigen-2/chondroitin sulfate proteoglycan 4 (NG2), the regulator of G-protein signaling-5 (RGS5), α-smooth muscle actin (αSMA), desmin, aminopeptidase N (CD13), endoglin (CD105), the adhesion molecule CD146, and many others (3). However, not all PC express every single marker; their expression is dynamic and varies between organs, developmental stages, activation/maturation state, and across individual microvascular networks. Neither are most of these markers found exclusively on PC (4). Moreover, it has been suggested that PC and vascular smooth muscle cells (VSMC) that surround larger vessels represent phenotypic variants of a continuous population of mural cells (5).

Initially, light and electron microscopy were the only techniques able to visualize them, and PC distinct from other perivascular cells could not be identified precisely. However, definition of PC by criteria that requires ultrastructural analysis for identification is not practical (6). Currently, at least two markers (positive reactivity to both PDGFR-β and NG2 being widely accepted) as well as morphology and cell location (in close contact with EC, embedded in the same basement membrane) are required to unequivocally distinguish PC from other mesenchymal cells (7). The use of transgenic mouse models fluorescently labeling PC [e.g., NG2-dsRed (8), the inducible NG2-CreERT2-eGFP (9), αSMA-GFP or αSMA-mCherry (10), and the double-transgenic nestin-GFP/NG2-DsRed mouse (11)] may be essential for studying the fate of PC under different conditions.

Pericytes have a well-known role in angiogenesis and vascular homeostasis, participating in guidance of the endothelial tip cells and vessel maturation and stabilization (12, 13). The initial stage of angiogenesis begins with PC–EC detachment and basement membrane degradation, followed by EC migration and proliferation and subsequent EC tube assembly and vessel stabilization by newly recruited PC (14). Recent studies support the notion that PC-mediated signaling may also be crucial for the growth phase of angiogenesis: PC may act as pioneers in the angiogenic sprout, creating pathways for guiding migrating EC (15).

Several signaling pathways have been characterized in the PC–EC crosstalk, including PDGF-B/PDGFR-β, TGF-β/ALK1/5, angiopoietin-1/Tie-2, and Jag1/Notch3 (16). Their role in the regulation of flow rate is more controversial (17), although discrepancies may be due to the lack of a clear definition of PC subpopulations (18). But PC not only provide structural support to EC, as it was classically assumed. Recently, several works have endowed PC with unexpected mesenchymal stem cell (MSC)-like properties. PC can express MSC markers and behave like MSC both *in vitro* and *in vivo*. Conversely, MSC have been attributed a perivascular origin (19) and can exhibit a PC-like behavior (20). They have also a role in cancer biology, where they participate in tumor angiogenesis and metastasis (21). However, PC have received far less credit as being immune regulators.

Rapidly expanding insights into their physiological and pathological functions have attracted the attention of many research groups. Beyond the field of angiogenesis research, PC have been mainly in the focus of neuroscientists, because of their central role in blood–brain barrier maintenance and the implication of their loss in diabetic retinopathy (13, 22). The PC “avatars” in the liver (hepatic stellate cells) and kidney (mesangial cells) have also received attention due to their role in fibrosis. Here, we aim to make these elusive cells mostly appealing for the community of immunologists.

## Are Pericytes Non-Professional Macrophage-Like Cells?

Pioneering studies suggested that PC do not represent mere bystanders in the inflammatory response, but display macrophage-like, non-professional antigen-presenting cell (APC) characteristics, suggesting possible participation in immune responses (23–25). In 1999, a comprehensive review summarized the knowledge about the potential role of brain PC (mainly of rodent origin) as macrophage-like cells (26). PC macrophage markers reported by different groups included CD4, major histocompatibility complex (MHC) class II molecules, CD45 (leukocyte-common antigen), Fc receptors, scavenger receptors, CD11b (alpha chain of the integrin Mac-1/CR3), the pan-macrophage marker CD68 (ED1), and the M2-polarized-specific marker CD163 (ED2) (23, 27–29). Among the macrophage-like properties of PC, pinocytosis and phagocytosis were documented. In this way, they could contribute to the removal of toxic cellular by products from the microcirculation. Very early *in vivo* studies (30, 31) had demonstrated accumulation of tracer particles in PC after systemic administration, which is suggestive of their phagocytic ability. Much later, phagocytic activity was confirmed in cultured primary rat PC using opsonized beads (23).

Macrophages are classical APC, which, in addition to MHC class II molecules, express co-stimulatory molecules such as CD80 and CD86. Non-professional APC that only express MHC class I molecules may function as APC after induction of MHC class II molecules expression by interferon-gamma (IFN-γ). Indeed, brain PC treated with IFN-γ expressed MHC class II molecules and acquired the capacity to present antigen to primed syngeneic T lymphocytes from rats immunized with myelin basic protein or ovalbumin (32). T cell proliferation was antigen specific and MHC class II dependent because an irrelevant antigen failed to induce incorporation of labeled thymidine and non-activated PC did not support it. The PC proliferative response was comparable to that produced by syngeneic APC and was dose and ratio dependent. Previously, cells referred to as smooth muscle/PC have been shown to selectively induce the Ag-specific activation of different Th1 clones, reflected by cell proliferation and production of IL-2 (33).

However, some of these pioneering findings have been questioned as clear-cut identification of PC was not provided, and the results might rather be attributed to perivascular macrophages (34). Controversy had already appeared in early studies using transmission electron microscopy that distinguished perivascular cells, which ingested carbon particles, from PC, which did not. The results of this study suggested that at least some perivascular cells remain distinct from PC (35).

More recent studies have reported that isolated porcine brain PC do not express MHC class II molecules under basal conditions, but IFN-γ can induce its mRNA (36) and protein expression (37). In a mouse model of PDGFR-β gain-of-function, activation of brain PC prompted the expression of immunoregulatory genes, including MHC class II molecules, Fcγ receptors and proteosome subunits as PSME1 (38). IFN-γ also upregulated CD68 mRNA, and both IFN-γ and TNF-α increased the phagocytosis of latex beads in SMA^+^, desmin^+^, CD90^+^, and NG2^+^ pig brain PC (36). On the other hand, PC phagocytic ability was attenuated by TGF-β1, possibly through downregulation of the scavenger receptors CD36, CD47, and CD68 (39).

However, in a recent work, pretreatment with different stimuli, including IFN-γ, failed to induce an APC-like phenotype in human *PC from various origins* (placenta, brain, and CD146^+^, CD105^+^ pluripotent stem cell-derived). PC constitutively expressed MHC class I but not MHC class II or the co-stimulatory molecules CD80 or CD86. As previously described, incubation of cultured PC with IFN-γ induced the expression of MHC class II by all types of PC. On the contrary, IFN-γ did not stimulate the expression of CD80 or CD86 and did not significantly affect the proliferation of CFSE-labeled CD4^+^ T cells in comparison with untreated PC (40).

In summary, more studies are needed to establish the role of PC as macrophage-like cells, once unequivocal identification is warranted.

## Pericyte Response to Pro-Inflammatory Cues

It is well known that activated EC can secrete a plethora of cytokines and chemokines that are important in potentiating inflammatory responses (41). Numerous *in vitro* studies have shown that EC secrete CXC (CXCL1, CXCL2, CXCL8, CXCL9, CXCL10, CXCL11), CC (CCL2, CCL3, CCL5, CCL7), and CX3C chemokines in response to various inflammatory stimuli. These studies correlate with *in vivo* studies demonstrating a role for these endothelial-derived chemokines in mediating leukocyte recruitment during various inflammatory conditions (42).

**Figure 1:**
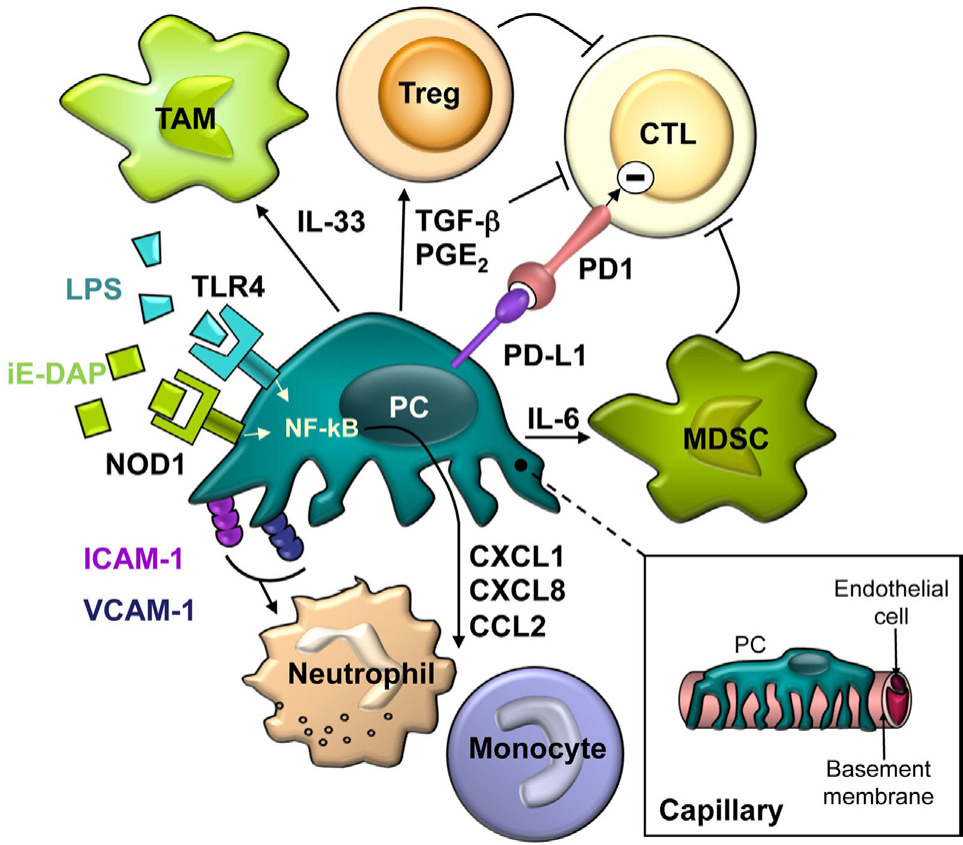
**The schematic drawing describes the close spatial relationship and the complex interactions between pericytes and different cells of the innate and adaptive immune system**.

Similarly, PC of multiple origins have been reported to secrete a plethora of chemokines and cytokines in response to pro-inflammatory stimuli released by professional innate immune cells, mainly TNF-α, IL-1β, and IFN-γ (Table 1). Interestingly, human PC have recently been shown to be much more responsive than human EC to IL-17 stimulation in producing pro-inflammatory molecules (43). Commonly upregulated pro-inflammatory factors include: CXCL8, IL-6, CCL2, CCL3, CCL5, CXCL1, and CXCL10. These cytokines and chemokines may be important in potentiating inflammatory responses by inducing cytokine secretion by other cells and recruiting immune cells to the site of inflammation. CXCL8 and CXCL1 bind to both receptors CXCR1 and CXCR2, expressed mainly by neutrophils. Of note, IL-17-stimulated PC not only overexpressed CXCL8 but also induced neutrophil synthesis of TNF-α, IL-1α, IL-1β, and CXCL8 (43). CCR2, the CCL2 receptor, is expressed mainly by monocytes. CXCR3 is the CXCL10 receptor, involved in Th1, CD8, and NK cell trafficking. CCL3 and CCL5 bind to CCR1 and CCR5 and modulate monocyte, macrophage, Th1, CD8, and NK cell migration (44). Therefore, all these cell types may be lured by activated PC to a site of inflammation (Figure 1).

**Table 1:** **Cytokines, chemokines, and adhesion molecules expressed by pericytes in response to pro-inflammatory stimuli**.

	Stimuli	Cell source	Reference
**Cytokine/chemokine**			
CCL2/MCP-1	LPS	HBP	(45)
	TNF-α, IL-1β, LPS	HBP	(47)
	LPS, TNF-α	HPP	(49)
	TNF-α	RBP	(51)
	TNF-α, IL-1β	HRP	(46)
	IL-1β	HBP	(48)
	IL-1β	HBP	(39)
	LPS, IFN-γ	HBP	(53)
CCL3/MIP-1α	LPS	MBP	(55)
	TNF-α	RBP	(51)
	TNF-α, IL-1β	HRP	(46)
CCL4	LPS	MBP	(55)
CCL5/RANTES	TNF-α	RBP	(51)
	HCMV	HBP	(54)
	TNF-α, IL-1β	HRP	(46)
CCL11/eotaxin	LPS	MBP	(55)
	TNF-α	HRP	(60)
CXCL1/GROα/KC	LPS	HBP	(45)
	LPS, TNF-α	HPP	(49)
	TNF-α	MMP	(61)
	TNF-α	RBP	(51)
CXCL10/IP-10	TNF-α, IL-1β, LPS	HBP	(47)
	TNF-α	RBP	(51)
	IFN-γ	HBP	(37)
CXCL11	HCMV	HBP	(54)
CX3CL1	IL-1α	HBP	(39)
G-CSF	LPS	MBP	(55)
	TNF-α, IL-1β	HRP	(46)
	TNF-α	HBP	(52)
GM-CSF	LPS	MBP	(55)
	TNF-α, IL-1β	HRP	(46)
IFN-γ	LPS	MBP	(55)
IL-1α	TNF-α	RBP	(51)
IL-1β	LPS	RLP	(60)
	High glucose	BRP	(62)
IL-2	TNF-α	RBP	(51)
IL5	TNF-α	RBP	(51)
IL-6	LPS	HBP	(45)
	TNF-α	RBP	(51)
	HCMV	HBP	(54)
	IL-17	HPP	(43)
IL-6 (Cont.)	TNF-α, IL-1β	HRP	(46)
	IL-1β	HBP	(48)
	LPS	MBP	(50)
	TNF-α	HBP	(52)
	TGF-β1	HBP	(39)
IL8/CXCL8	LPS, HMGB1	HBP	(45)
	LPS, TNF-α	HPP	(49)
	HCMV	HBP	(54)
	TNF-α, IL-1β	HRP	(46)
	LPS, TNF-α, IL-1β	PBP	(56)
	IL-1β	HPP	(57)
	C12-iE-DAP	HBP	(58)
	IL-1β	HBP	(48)
	TNF-α	HBP	(52)
	IL-1β	HBP	(39)
	TNF-α	HPP	(59)
	IL-17	HPP	(43)
IL-10	LPS	MBP	(55)
IL-12	LPS	MBP	(55)
IL-13	LPS	MBP	(55)
IL-17	TNF-α	RBP	(51)
MIF	LPS, TNF-α	HPP	(49)
TNF-α	LPS, TNF-α	MBP	(55)
	High glucose	BRP	(62)
**Adhesion molecule**			
ICAM-1	TNF-α, IFN-γ	RBP	(32)
	LPS	HBP	(45)
	TNF-α	MMP	(61)
	TNF-α, LPS	HPP	(49)
	TNF-α, IFN-γ	HBP	(63)
	IL-1β	HPP	(57)
	High glucose	BRP	(62)
	IL-1β	HBP	(48)
	TNF-α	HPP	(59)
	TNF-α, IFN-γ	HPP	(64)
	LPS, IFN-γ	HBP	(53)
VCAM-1	TNF-α	RBP	(32)
	LPS	HBP	(45)
	TNF-α	HBP	(63)
	TNF-α	HPP	(59)

*BRP, bovine retinal pericytes; HBP, human brain pericytes; HCMV, human cytomegalovirus; HPP, human placental pericytes; HRP, human retinal pericytes; MBP, mouse brain pericytes; MMP, mouse muscle pericytes; PBP, pig brain pericytes; RBP, rat brain pericytes; RLP, rat lung pericytes*.

Chemokines produced by PC also have a key role in inflammation-associated angiogenesis. CXCL1 and CXCL8 belong to the CXC chemokine subgroup with glutamic acid–leucine–arginine (the ELR motif) immediately proximal to the CXC sequence, which promote the migration and proliferation of CXCR1- and CXCR2-expressing EC (65). In addition to the ELR+ CXC chemokine family, CCL2 is the best known CC chemokine mediator of neovascularization. This pattern of chemokine production may shed new light on PC–EC interactions, suggesting a dual role for PC according to their activation status. In physiological conditions, PC are necessary to maintain a quiescent stable endothelium; however, activated PC could act as drivers of angiogenesis during inflammatory processes.

Increased vascular permeability is a hallmark of inflammation, and PC may play a role in this response. It has been shown that TNF-α, IL-1β, and IFN-γ promote the expression of inducible nitric oxide synthase (iNOS) by porcine (36) and rat (51) brain PC. The generation of NO by PC can act in both an autocrine and paracrine manner as a relaxing factor, which leads to vasodilation. In addition, an iNOS-independent pathway by which lung PC contractility is regulated by lipopolysaccharide (LPS) has been suggested (66). VEGF modifies the contractile response of lung PC, and this mechanism may play a role in the increased permeability demonstrated in inflammatory conditions (67). PC also upregulate cyclooxygenase-2 (COX-2), responsible for production of inflammatory prostaglandins, and generate reactive oxygen and nitrogen species after the stimulation with TNF-α, IL-1β, or IFN-γ (36). In another study, microarray analysis of PDGFRβ^+^, SMA^+^ human brain PC treated with IFN-γ and IL-1β revealed widespread changes in gene expression including upregulation of interleukins, chemokines, adhesion molecules, *PTGS2/COX2*, and *SOD2* (47). Increased superoxide dismutase expression by PC could confer tolerance to oxidative stress in the inflammatory context.

Aside from participating in the onset of inflammatory responses, autolimiting mechanisms have also been identified in PC. The transcription factor C/EBPδ is induced in a concentration- and time-dependent fashion in PDGFRβ^+^, SMA^+^, and NG2^+^ human brain PC by IL-1β, limiting PC production of CCL2 and thereby preventing further inflammatory responses (48).

All these works support the potential of PC to take part in immunological responses under inflammatory conditions (36). However, their role is complex: Pdgfrβ^+/−^ mice showed an age-dependent progressive loss of PC coverage in the brain associated with neutrophil infiltration and expression of several inflammatory factors (TNF-α, IL-1β, IL-6, CCL2) and ICAM-1 (68).

## PC as Sentinels of the Innate Immunity

Several classes of receptors, collectively termed pattern-recognition receptors (PRR) are responsible for sensing microorganisms and endogenous molecules released during cell injury. These germ line-encoded PRR recognize conserved pathogen-associated molecular patterns (PAMPs) and danger-associated molecular patterns (DAMP). Five families of PRR have been identified in mammals, of which toll-like receptors (TLRs) and NOD-like receptors (NLRs) are the most studied and characterized (69). Beyond the passive role of PC in the amplification of inflammatory responses above described, expression of functional PRR suggests that brain PC may directly contribute to the onset of innate immune responses.

Human EC are known to express several TLRs, whereas inflamed endothelium has significant upregulation of TLR2 (receptor for di/triacyl lipopeptides) and TLR4 (LPS receptor) (70, 71). However, expression of these receptors in PC has been scarcely addressed. In a series of pioneering works by Edelman et al., the authors demonstrated TLR4 upregulation (72), increased vessel permeability (73), and production of IL-1β (60) in rat lung PC treated with LPS, suggesting an active role of PC in inflammation. Subsequently, the release of NO and several cytokines and chemokines by SMA^+^, CD13^+^ mouse brain vascular PC in response to LPS was reported (55). More recently, we have documented the expression of TLR4 by human brain PC and their responsiveness to both LPS and the endogenous ligand HMGB1 (45). In this study, the transcriptional profile of LPS-treated PC was characterized using DNA microarrays. Shortly after, Jansson et al. confirmed the ability of human brain PC to respond to different pro-inflammatory stimuli, including LPS (47).

Pattern-recognition receptors signaling in innate immune cells results in the production of a variety of pro-inflammatory molecules. In LPS-treated human brain PC, six genes were found to be upregulated more than 15-fold: *CXCL10, CCL20, CXCL8, CXCL1, IL-6*, and *CCL2* (45) (Table 1). Interestingly, these data corroborated a different capacity in synthesis of pro-inflammatory factors between vascular cells and leukocytes in response to PAMPs or analogs. It had been described that vascular VSMC and EC cells produce 10- to 100-fold more IL-6, CXCL8, and CXCL10, whereas leukocytes are nearly exclusive producers of IFN-γ and TNF-α, at least in humans (74). Intriguingly, LPS induces in human coronary artery EC mRNA expression of *IL-1*α, *IL-1*β, and *TNF-*α, but no release of the proteins (75). Similarly, a significant upregulation of *IL-1*α and *IL-1*β mRNA in human brain PC was observed, but expression at protein level could not be detected (45). The profiles of cytokines and chemokines released by human and mouse brain PC in response to LPS also differ considerably, supporting major species differences in vascular cell immunological functions, as highlighted by Pober and Tellides (76). Indeed, it is controversial how closely the gene-expression patterns in mouse models recapitulate those in human inflammatory conditions (77, 78).

A recent report described that LPS-induced secretion of certain inflammatory cytokines/chemokines such as CCL2 and CXCL1 by HUVEC was inhibited by the axon guidance molecule Slit2 and its receptor Robo4 pathway (79). Previously, it had been shown that Robo4-dependent Slit2 signaling reduces the endothelial hyperpermeability induced by LPS *in vivo* (80). Interestingly, we described the expression of Robo4 in human brain PC and their response to Slit2 (81), which inhibited spontaneous and PDGF-B-induced migration of PC. It remains to be addressed whether the Slit2-Robo4 axis also modulates the inflammatory response in human brain PC.

NOD1 and NOD2, the prototypical members of the NLR family of PRR, mediate the cytoplasmic recognition of peptidoglycan (PGN) fragments (82). NOD1 is expressed in a variety of cell types of both hematopoietic and non-hematopoietic origin, including EC, where it has been shown to be critical in sensing pathogens (83) and mediating vascular inflammation (84). The expression of these PRR in PC had not been addressed, but a recent report by our group showed that NOD1 is functionally expressed by PC, whereas NOD2 expression is barely detectable. The NOD1 agonist C12-iE-DAP induced *IL-6* and *CXCL8* gene expression by PC as well as their release into culture supernatant. Moreover, the synergistic effect of NOD1 and TLR4 agonists on the induction of *CXCL8* was demonstrated (58).

Recently, it has been reported that human brain primary PC cultures contain at least two functionally distinct CD73^+^ cell types: one being the more proliferative, CD90^+^ (Thy-1) cells, and CD90^−^ cells, which show a greater inflammatory response to LPS and IFN-γ stimulation (53). In fact, the primary human brain PC we used to characterize the response to LPS, HMGB1, and C12-iE-DAP expressed canonical high levels of PDGFR-β, NG2, CD13, CD73, and CD105 but unexpectedly lacked CD90, as assessed by flow cytometry (81). It will be interesting to learn more about the biological significance of CD90 and the discrete roles of these PC subpopulations in physiological and pathological conditions.

## Pericyte Control of Immune Cell Trafficking

Leukocyte trafficking to target tissues is orchestrated by adhesion molecules and chemokines that stabilize dynamic interactions between immune cells and EC. While PC have long been disregarded as players in this process, accumulating evidence have shed light on the significance of these cells as regulators of leukocyte recruitment to inflammation sites. This new understanding has been enabled by advances in microscopy techniques and the generation of genetically fluorescent animal models. Intravital imaging studies have revealed the events following leukocyte transendothelial migration *in vivo*: to fully exit venular walls, infiltrating cells must breach the basement membrane and cross the PC sheath to enter the perivascular space, which creates a special milieu that controls the behavior and fate of infiltrated immune cells (85, 86).

Recent works have demonstrated that PC facilitate neutrophil transmigration in a model of TNF-α- or IL-1β-stimulated mouse cremaster muscle *in vivo* (61, 87). Namely, PC were observed to provide a substrate for neutrophils creeping along their processes (“abluminal crawling”) to gaps between adjacent PC which are enlarged in inflamed tissues and used as exit points. This response was mediated through the interaction of PC-expressed ICAM-1 with neutrophil Mac-1 and LFA-1 (61). Previous studies by the same group had shown that these gaps were aligned with regions of low densities of the extracellular matrix proteins collagen IV and laminin 10 in the basement membrane (low-expression regions, LERs) (88–90). PC relaxation rather than contraction contributes to the opening of the gaps between PC and to the widening and thinning of LERs, facilitating neutrophil extravasation (87).

Moreover, capillary and arteriolar NG2^+^ PC can “instruct” extravasating neutrophils and monocytes with migratory cues after exiting through postcapillary venules covered by NG2^−^ PC (49). In response to inflammatory mediators, NG2^+^ PC upregulated expression of ICAM-1 and released the chemoattractant MIF, which directed interstitial leukocyte trafficking. In this setting, PC-driven migration of monocytes was mediated mainly by MIF and CCL2, whereas neutrophil migration involved MIF and CXCL8. These interactions with the abluminal face of NG2^+^ PC were crucial for the efficient navigation of cells of the innate immune system and enhanced the ability of the latter cells to screen the interstitial space for damaged tissue and to execute their effector functions at foci of sterile inflammation.

Significantly, the *in vitro* coculture of human umbilical vein EC and CD90^+^, NG2^+^ human placental PC indicated that transendothelial migration itself, which increases neutrophil Mac-1 surface expression, can prime neutrophils for enhanced interactions with PC (57). Another *in vitro* model showed that porcine brain PC are able to chemoattract neutrophils by CXCL8 after stimulation with LPS, TNF-α, or IL-1β (56). Remarkably, LPS-treated human brain PC showed a strong increase in the expression of both ICAM-1 and VCAM-1 which promoted a significant increase in adhesion of peripheral blood lymphocytes to human brain PC (45). Notably, VCAM-1/VLA-4 interaction was proposed more than 20 years ago as a mechanism mediating T cell–human brain PC crosstalk (63). In a much more recent study, PC have been shown to control mature T cell transmigration across the endothelium from the thymus into circulation (91).

Collectively, these findings showed that interactions of PC with different types of leukocytes modulate their trafficking through vessel walls. The impact of such interactions on the phenotype and activation state of immune cells requires further investigations.

## Modulation of Adaptive Immune Responses by PC

If PC can regulate T cell trafficking, it is obvious that they are also positioned to modulate T cell activation (76) (Table 2). Human EC have been considered amateur APC, since they lack expression of key co-stimulatory molecules such as CD80 and CD86, as it has been reported for PC. But PC also display higher levels of PD-L1 and PD-L2, ligands for the inhibitory immune checkpoint molecule PD1 expressed by activated T cells. In agreement with these observations, IFN-γ-treated MHC class II^+^ human placental PC, unlike EC, cannot stimulate resting allogeneic CD4 T cell proliferation or cytokine production. Instead, coculture with PC renders T cells anergic (64). These placental PC express characteristic markers NG2, CD90, CD146, and SMA. Like PC, the ontogenetically related MSC have been reported to inhibit T cell proliferation (92).

**Table 2:** **Modulation of T cell activation and antitumoral immune response by pericytes**.

Pericyte type/origin	*In vitro* effect	Tumor model	*In vivo* effect	Reference
HBP	T cell adhesion, VCAM-mediated	N/A	N/A	(63)
HPP	T cell anergy	N/A	N/A	(64)
HRP, MRP	T cell inhibition, PDL1/IL-10-mediated	N/A	N/A	(93)
HPSC-derived PC, HBP, HPP	T cell hyporesponsivenes, induction of Tregs, PD-L1/TGFβ-mediated	N/A	N/A	(40)
C3H10T1/2-*in vitro* differentiated, tumor-conditioned PC; B16 tumor-derived PC	CD4+ T cell anergy, RGS5- and IL-6-dependent	B16 mouse melanoma	N/A	(94)
HBP	T cell anergy, PGE2-, NO-, HGF, TGFβ-mediated	Human malignant glioma	N/A	(95)
PDGF-B ret/ret mouse model (pericyte-deficient)	N/A	B16 melanoma, LLC mouse lung cancer	Recruitment of T-cell suppressive MDSC, IL-6 mediated. Increased tumor growth and metastasis	(96)
Rgs5−/− mouse model	N/A	RIP1-Tag5 (insulinoma) × Rgs5−/− mouse model	Vascular normalization and enhanced infiltration of CD8+ T cells. Increased survival	(97)
FVB/N mice	N/A	NT-2 mouse breast cancer	Increased infiltration of CD8+ cells after vaccination against pericyte antigens. Delayed tumor growth	(98)
C57BL/6, HDD (HLA-A2 transgenic) mice	N/A	MC38 mouse colon carcinoma, B16 melanoma	Increased infiltration of CD8+ cells after vaccination against pericyte antigens. Tumor eradication	(99)
SCID, C57BL/6, C57BL/6 IL-33−/− mice. Isolated LMP	PDGF-BB-induced IL-33 expression in LMP. Increased migration of IL-33-primed macrophages	*pdgfb*-shRNA A431 human epidermoid carcinoma, *pdgfb*-overexpressing murine T241 fibrosarcoma and LLC cells	Recruitment of TAM, IL-33 mediated. Metastasis promotion	(100)

*HBP, human brain pericytes; HPP, human placental pericytes; HPSC, human pluripotent stem cells; HRP, human retinal pericytes; LLC, Lewis lung carcinoma; LMP, lung mouse pericytes; MDSC, myeloid-derived suppressor cells; MRP, mouse retinal pericytes; TAM, tumor-associated macrophages*.

Retinal PC also have immunosuppressive properties, and coculture with activated T cells decreased proliferation and IFN-γ and TNF-α production in a dose-dependent manner (93). Both cell–cell contact and soluble factors are involved in retinal PC-mediated T cell inhibition, since it was decreased by the addition of blocking anti-PD-L1 and anti-IL-10 antibodies, and in transwell experiments. Interestingly, retinal PC protected EC from T cell-induced death, suggesting that their loss under hyperglycemic conditions favors retinal inflammation and contributes to the pathogenesis of diabetic retinopathy.

Characterization of different subsets of lymph node stromal cells (LNSC) identified a novel gp38- and CD31-double-negative population. These cells expressed high levels of autoimmune regulator (AIRE) and showed a strong response to inflammation by upregulating peripheral tissue-restricted antigen expression. Of interest, double-negative LNSC upregulated PD-L1 in PolyI:C-treated mice, suggesting a potential contribution to shaping the T cell repertoire and peripheral tolerance (101). In a subsequent study, transcriptional profiling of LNSC analyzing each subset’s expression signature identified double-negative LNSC as PC able to respond to inflammatory or infectious triggers (102).

In another study, human brain or placental PC and PC derived from human pluripotent stem cells (hPSC) mediated a significant increase in the frequency of allogeneic CD25 high FoxP3^+^ regulatory T cells (Tregs) when cocultured with non-activated peripheral blood T cells. PD-L1/2 expression and secretion of TGF-β by hPSC PC directly regulated generation of Tregs favoring allostimulation of Tregs over T cell activation, suggesting that hPSC PC could be applied to allogeneic cell therapy in the clinic, not only without provoking immediate immune responses but also actively modulating suppressive immunity (40).

In summary, PC are not only players in innate immunity and inflammation but they can also participate, at least under certain circumstances, in adaptive immunity.

## Immune Regulation by PC in the Tumor Microenvironment

The role of stromal cells in the tumor microenvironment (TME) has attracted great interest, and PC are *bona fide* components of the TME, although its coverage of tumor microvasculature is controversial (103–105). Undoubtedly, the immunomodulatory properties of PC may have an impact in the context of antitumor immune responses. Along with PD-L1 and PD-L2 expression, PC from normal human brain and human malignant glioma (both PDGFRβ^+^, desmin^+^, SMA^+^, and NG2^+^) have been shown to secrete various factors with immunosuppressive properties, such as NO, PGE2, and TGF-β (95). Not surprisingly, previous *in vitro* results have a correlation with the antitumor immune response *in vivo*. PC derived from subcutaneously implanted B16 or CT26 tumors expressed, unlike their normal counterparts, MHC class II and CD80 molecules (94). In this work, PD-L1 expression was upregulated in normal PC after culture in tumor-conditioned media. Tumor-derived PC, but not normal PC, negatively influence CD4^+^ T cell activation and proliferation *in vitro*, and promote anergy in OVA-specific cells in culture. This immunoregulatory capacity was dependent on TME-induced RGS5 expression and IL-6, and could help tumors to evade immune responses (94). Similarly, PC isolated from human malignant glioma were equally capable of suppressing allogeneic or mitogen-activated T cell responses *in vitro* through the production of PGE2, TGF-β, and NO. Moreover, CD90^+^ PDGFR-β^+^ perivascular cells accumulated in human gliomas with increasing degree of malignancy and negatively correlated with the presence of blood vessel-associated leukocytes and CD8^+^ T cells (95).

In line with these findings, PC targeting has shown antitumor effects *in vivo*. A study with the RIP1-Tag5 mouse model of pancreatic carcinoma showed that deletion of the Rgs5 gene induced changes in the vasculature and enhanced infiltration of CD8^+^ T cells in tumors after adoptive transfer. As a consequence, the immune-mediated tumor rejection was exacerbated, resulting in improved survival of tumor-bearing mice (97). Notably, RGS5 is also overexpressed in tumor PC in this model, similar to what has been documented for several human tumors, including kidney, liver, and head and neck cancers (97, 106).

Tumor PC have also been targeted using vaccination approaches. In a breast carcinoma model, immunization with a *Listeria monocytogenes*-based vaccine against NG2 was shown to promote tumor infiltration of CD8^+^ T cells and tumor regression (98). Indeed, vaccination with peptides derived from PDGFR-β and RGS5 were also effective in preventing HLA-A2^−^ colon carcinoma (MC38) establishment or resulted in the regression of tumors in HLA-A2 transgenic mice (99). Effective vaccination resulted in profound infiltration of tumor lesions by CD8^+^ cells and supported the idea that targeting tumor PC can alleviate local immunosuppression.

However, the role of PC in TME is complex, as they may contribute to different cancer hallmarks beyond immune evasion (107), and consequently their role as potential targets in cancer immunotherapy approaches should be carefully evaluated. In the PDGF-B (ret/ret) mouse model, PC deficiency produced defective tumor vasculature, resulting in a more hypoxic microenvironment. Hypoxia promoted IL-6 upregulation in the malignant cells and increased transmigration of myeloid-derived suppressor cells (MDSC) in experimentally induced tumors. MDSC accumulation in tumors led to increases in tumor growth, whereas restoring the PC coverage in tumors abrogated the increased MDSC trafficking to PC-deficient tumors (96). Though, another study reported that IL-33 produced by PDGF-B-stimulated PC promoted metastasis through recruitment of tumor-associated macrophages in several human and mouse graft tumor models (100). Further extensive studies will be required to understand the crosstalk of PC with immune cells different from T cells.

## Conclusion and Perspectives

Pericytes have demonstrably been shown to possess an immunological role beyond their structural role in the microvasculature. PC can respond to a series of pro-inflammatory stimuli and are also able to discriminate between several types of danger and mount a complex secretory response: upon PAMP engagement, PRR trigger intracellular signaling cascades ultimately culminating in the expression of a variety of pro-inflammatory molecules. At the same time, PC overexpress adhesion molecules that guide and instruct innate immune cells after transendothelial migration. Moreover, PC are implicated in shaping adaptive immunity, with several studies that point to an immunosuppressive role. This role may have an impact on the antitumor immune response, since PC are constituents of the TME. A better understanding of the mechanisms by which PC communicate with their neighboring cells and modulate immune responses in tumors can be expected to yield exciting new insights as well as help in the development of new therapeutic targets with important implications for cancer immunotherapy.

## Author Contributions

All authors listed have made substantial, direct, and intellectual contribution to the work and approved it for publication.

## Conflict of Interest Statement

The authors declare that the research was conducted in the absence of any commercial or financial relationships that could be construed as a potential conflict of interest. The reviewer X-YW and handling Editor declared their shared affiliation, and the handling Editor states that the process nevertheless met the standards of a fair and objective review.
